# Schistosomiasis Control Using Piplartine against *Biomphalaria glabrata* at Different Developmental Stages

**DOI:** 10.1371/journal.pntd.0002251

**Published:** 2013-06-06

**Authors:** Ludmila Nakamura Rapado, Alessandro de Sá Pinheiro, Priscila Orechio de Moraes Victor Lopes, Harold Hilarion Fokoue, Marcus Tullius Scotti, Joaquim Vogt Marques, Fernanda Pires Ohlweiler, Sueli Ivone Borrely, Carlos Alberto de Bragança Pereira, Massuo Jorge Kato, Eliana Nakano, Lydia Fumiko Yamaguchi

**Affiliations:** 1 Laboratório de Parasitologia, Instituto Butantan, São Paulo, Brazil; 2 Instituto de Ciências Biomédicas, Universidade de São Paulo, São Paulo, Brazil; 3 Laboratório de Ensaios Biológicos e Ambientais, Instituto de Pesquisas Energéticas e Nucleares, IPEN/CNEN, São Paulo, Brazil; 4 Laboratório de Química de Produtos Naturais, Universidade de São Paulo, São Paulo, Brazil; 5 Universidade Federal da Paraíba, Centro de Ciências Aplicadas e Educação, Campus IV, Rio Tinto, Brazil; 6 Laboratório de Malacologia - Divisão de Programas Especiais - Superintendência de Controle de Endemias, São Paulo, Brazil; 7 Instituto de Matemática e Estatística, Universidade de São Paulo, São Paulo, Brazil; National Institute of Parasitic Diseases, Chinese Center for Disease Control and Prevention, China

## Abstract

**Background:**

Schistosomiasis is one of the most significant diseases in tropical countries and affects almost 200 million people worldwide. The application of molluscicides to eliminate the parasite's intermediate host, *Biomphalaria glabrata*, from infected water supplies is one strategy currently being used to control the disease. Previous studies have shown a potent molluscicidal activity of crude extracts from *Piper* species, with extracts from *Piper tuberculatum* being among the most active.

**Methods and Findings:**

The molluscicidal activity of *P. tuberculatum* was monitored on methanolic extracts from different organs (roots, leaves, fruit and stems). The compounds responsible for the molluscicidal activity were identified using ^1^H NMR and ESIMS data and multivariate analyses, including principal component analysis and partial least squares. These results indicated that the high molluscicidal activity displayed by root extracts (LC_50_ 20.28 µg/ml) was due to the presence of piplartine, a well-known biologically-active amide. Piplartine was isolated from *P. tuberculatum* root extracts, and the molluscicidal activity of this compound on adults and embryos of *B. glabrata* was determined. The compound displayed potent activity against all developmental stages of *B. glabrata*. Next, the environmental toxicity of piplartine was evaluated using the microcrustacean *Daphnia similis* (LC_50_ 7.32 µg/ml) and the fish *Danio rerio* (1.69 µg/ml). The toxicity to these organisms was less compared with the toxicity of niclosamide, a commercial molluscicide.

**Conclusions:**

The development of a new, natural molluscicide is highly desirable, particularly because the commercially available molluscicide niclosamide is highly toxic to some organisms in the environment (LC_50_ 0.25 µg/ml to *D. similis* and 0.12 µg/ml to *D. rerio*). Thus, piplartine is a potential candidate for a natural molluscicide that has been extracted from a tropical plant species and showed less toxic to environment.

## Author Summary

Schistosomiasis is a disease caused by parasitic worms of several species of genus *Schistosoma* that affects almost 200 million people mostly common in Asia, Africa and South America. The transmission is carried out by the parasitic larvae hosted in fresh water snails of the genus *Biomphalaria*. Considering the socioeconomic importance of this disease, the management of the snail population in the lakes and fresh water sources is one strategy to control the schistosomiasis. Nowadays, one synthetic compound, niclosamide, is available, but it is considered toxic to other organisms in the environment. Thus in this work piplartine was evaluated as a new active natural molluscicide extracted from a tropical plant. In addition a fish *Danio rerio* and a microcrustacean *Daphnia similis* were used as model organisms to evaluate the environmental toxicity risk of piplartine that was less toxic compared to niclosamide in the experimental conditions.

## Introduction

Schistosomiasis is a tropical disease caused by parasitic worms of the genus *Schistosoma* and is found predominantly in areas without sanitization or clean water, including regions of Africa, South Asia and Central and South America. Presently, this disease affects an estimated 200 million people worldwide [1], [2]. In the Americas, the only human schistosome is *Schistosoma mansoni*, which uses mollusks of the genus *Biomphalaria* as its intermediate host. One strategy used to control schistosomiasis is the management of snail populations in lakes and rivers using synthetic molluscicides. Presently, niclosamide (Bayluscide, Bayer, Leverkusen, Germany) is the only commercially available molluscicide that has been recommended by the World Health Organization (WHO) for large-scale use in Schistosomiasis Control Programs [3]. However, niclosamide is also toxic to non-target organisms. Furthermore, the application of niclosamide is costly, and this drug does not prevent recolonization of sites by surviving snails, which could lead to the selection of molluscicide-resistant populations [4]–[6]. Due to these disadvantages, the WHO is eager to find alternative drugs to facilitate schistosomiasis control; among these efforts is ongoing research on plant molluscicides, which have been considered, in several cases, as potential candidates due to their accessibility, structural diversity, low cost and possible rapid biodegradation [7].

Members of the Piperaceae family have been widely studied as a source of secondary metabolites with biological activity; among these species, *Piper tuberculatum* extracts, or their isolated compounds, have shown a diverse range of biological activities, such as insecticidal and fungicidal properties [8]–[11]. In a previous study, *P. tuberculatum* crude extracts showed molluscicidal activity against *B. glabrata* adult snails [12]. Additionally, many researchers have emphasized that the amides present in *P. tuberculatum* could be responsible for the antifungal, antitumor, antiparasitic and antiproliferative activities assigned to this species [10], [13]–[16].

In this study, the primary compound responsible for the molluscicidal activity attributed to *P. tuberculatum* crude extracts from different organs was identified by ^1^H NMR and ESIMS data, combined with principal component analysis (PCA). Additionally, partial least squares (PLS) analysis was performed to provide quantitative analysis and to confirm the pattern visualized in the PCA. The amides piplartine, piperine, piperlonguminine and pellitorine isolated from different organs were evaluated for molluscicidal activity on *B. glabrata* adults and embryos. The results obtained associating the multivariated analysis (PCA and PLS) with chemical composition and molluscicide activity revealed piplartine as principal amide responsible for the activity in *P. tuberculatum*. The acute toxicity of piplartine was also evaluated using validated ecotoxicological assays in the daphnid *Daphnia similis* and the fish *Danio rerio*.

## Materials and Methods

### Ethics Statement

This study was performed in strict accordance with the recommendations by the Aquatic ecotoxicology – Acute toxicity – Test with fish according to ABNT NBR (Brazilian Assocn. of Tech. Stds.) 15088 (norms related to evaluation of the acute toxicity in *Danio rerio* and *Pimephales promelas* of samples from effluents, superficial or subterranean water supplies and chemical substances soluble or dispersed in water). The protocol was approved by Comissão de Ética no uso de animais do Instituto Butantan (CEUAIB), São Paulo, Brazil (Permit Number: CEUAIB 434/07).

### Plant Material

*P. tuberculatum* Jacq. was collected from the Chemistry Institute at University of São Paulo, and the botanical classification was performed by Dr. Elsie Franklin Guimarães (Instituto de Pesquisas Jardim Botânico do Rio de Janeiro). A voucher specimen (Kato-169) was deposited in the herbarium of the same institute.

### Preparation of *P. tuberculatum* Extract

The roots, stems, leaves and fruits of *P. tuberculatum* were dried in an oven at 45°C. The organs were then ground, and the powdered materials were extracted with methanol at room temperature (25–27°C) three times and filtered. Extracts were evaporated to dryness under vacuum in a rotaevaporator and stored. A stock solution containing 1,000 µg/ml of each extract was prepared by suspending 10 mg of extract in 0.1 ml of 99.9% dimethylsulphoxide (DMSO; Aldrich, Milwaukee, Wisconsin, USA) and bringing the volume to 100 ml with dechlorinated water. Stock solutions were diluted with dechlorinated water for use in assay solutions.

### ^1^H NMR and Mass Spectra Analysis for PCA and PLS

NMR analysis was performed using 20 mg of *P. tuberculatum* extracts obtained from different organs of the plant. The samples were dissolved in 800 µl CDCl_3_ (99.8%, Cambridge Isotopes Laboratories TM) containing 0.05% of tetramethylsilane as an internal standard. The ^1^H NMR spectra were obtained with a Bruker DPX 200 MHz 5 mm probe. Each spectrum consisted of 256 scans and 300 k data points, with a pulse width of 8.0 µs (30°) and relaxation delay of 2.0 s. All spectra data were subjected to Fourier transformation using the program MestReC (version 4.8.6.0, Mestrelab) and had line broadening of 0.4 Hz. Spectra signals were integrated in regions of equal width (0.02 ppm) corresponding to the region δ 0.5–10.00. The signals corresponding to each amides were assigned based on published data [10].

ESIMS analyses were performed in a Quattro II triple quadrupole mass spectrometer (MS) (Micromass, Manchester, UK). First, the samples were prepared by dissolving the crude extract in MeOH at a concentration of 1 mg/ml. The electrospray positive ionization mode was employed with a capillary voltage of 4.5 kV, skimmer of 50 V and nitrogen gas flow of 250 and 30 l/h. Samples were injected directly into the MS using a mobile phase flow of 50 µl/min (MeOH∶H_2_O 1∶1), and the data were processed with MassLynx (Micromass) version 3.2. The molecular mass to charge ratios (*m/z*) of each amides were determined calculating the molecular formulae of each compound according to previous studies [10]. The quasi-molecular ions detected for piplartine (C_17_H_19_NO_5_, MW 317) were corresponding to its sodium adduct [M+Na]^+^ 340, [M+Na+1]^+^ 341 and its fragments at *m/z* 221 and 222. The *m/z* detected for pellitorine was 224 and 225 corresponding to its molecular mass (C_14_H_25_NO). The piperine, dihydropiperine and dihydropiperlonguminine were detected by *m/z* 286, 288 and 276 corresponding to their molecular mass C_17_H_19_NO_3_, C_17_H_19_NO_3_ and C_16_H_21_NO_3_, respectively.

### PCA and PLS Analysis

The PCA and PLS analysis were performed using ^1^H NMR, ESIMS and molluscicide activity data of methanolic extracts from different organs of *P. tuberculatum*.

To minimize the potential lack of reproducibility that is associated with both the headspace generation process and the response of the mass detector, the signals generated (raw data) were subjected to area normalization, in which the area under the curve becomes equal for all spectra [17]. The same normalization process was applied to ^1^H NMR to reduce systematic variations due to intensity scaling effects resulting from variations in the total concentrations of solutes between samples. LC_50_ values (µg/ml) were submitted to the standard score process, in which the mean is subtracted from the variable values, and the resultant values are divided by the standard deviation. To perform the PCA and PLS analyses, each variable (i.e., each ^1^H NMR integrated region and intensity of *m/z* mass to charge ratios in the mass spectra of each sample) was subtracted by the variable mean; this process ensured that all results would be interpretable in terms of variation from the mean. Leave-one-out cross validation was used to determine the robustness of the generated PLS model.

### Isolation of Amides

The amides pellitorine, piperlonguminine and piperine were purified as previously described [10]. Methanolic extracts from different organs of *P. tuberculatum* were submitted to successive column chromatography using silica gel and a gradient of solvents at increasing polarity. The NMR data indicated that the composition of root crude extract was accounted for 92% piplartine; thus, this extract was submitted to recrystallization in MeOH to obtain pure piplartine. Consistent with common recrystallization protocols, 200 mg of crude extract from roots was dissolved in 3 ml of hot MeOH and recrystallized, yielding 140 mg of pure piplartine. Piplartine was identified using ^1^H NMR analysis (Bruker DPX 200 MHz) in CDCl_3_ (99.8%, Cambridge Isotopes Laboratories, Inc.) and compared with authentic sample available [10].

### Biological Assays

Tests were performed according to the methodology recommended by the WHO [5], [7]. Adults and egg masses of *B. glabrata* (Say, 1818) were obtained from a Belo Horizonte population (MG, Brazil) and reared under laboratory conditions for several years, with fresh lettuce *ad libitum* to maintenance and a balanced ration during the assay.

In all assays, both positive and negative controls were used to examine the susceptibility of the organisms under the assay conditions. The commercially available molluscicide niclosamide was used in the positive control group; the negative control group received dechlorinated tap water containing 1% DMSO.

### Molluscicidal Activity

Snails with 10–18 mm of shell diameter were exposed to *P. tuberculatum* extracts and amides (piplartine, piperlonguminine, pellitorine and piperine) for 24 h at 25°C±2°C. After exposure, the snails were washed and observed daily for 7 days, and the death rate was recorded. For *P. tuberculatum* root, stem, leaf and fruit extracts, concentrations less than 1000 µg/ml were evaluated, and amides were evaluated at concentrations less than 20 µg/ml. The LC_90_ and LC_50_ values were determined. Ten animals were used per concentration and experiments were repeated three times.

### Ovicidal Activity

Plastic sheets served as the substrate for oviposition, and small circles with one egg mass attached were excised. Five egg masses at the blastula, gastrula, trocophore and veliger stages [18] were exposed to piplartine, pellitorine, piperlonguminine or piperine at concentrations below 20 µg/ml for 24 h to determine the LC_90_ and LC_50_ values. Following the exposure, the egg masses were washed and observed for mortality and malformations daily for 7 days using stereomicroscopy. Assays were repeated three times with approximately 100 embryos for each concentration.

### Ecotoxicity Assays

#### Microcrustacean

*D. similis* (Cladocera, Crustacea) were obtained from the Biological and Environmental Research Laboratory, Institute of Nuclear and Energy Research, Brazil. Daphnids (25 adults/l) were maintained in 2 l glass flasks in a chamber with controlled temperature (20±2°C) and a light intensity of 1,000 lux under a 16 h period of light. Daphnids were grown in natural water with an adjusted total water hardness of 46 mg/l CaCO_3_ (pH 7.0±0.5). The organisms were fed daily with a suspension of *Pseudokirchneriella subcaptata* green alga (3.6×10^5^ cells/ml), supplemented with a mixture of yeast and fish meal.

The acute toxicity assays with *D. similis* were performed according to ABNT NBR 12713 [19], [20] and in conformity with OECD (2004). Five neonates (6–24 h old) were placed in 50 ml glass beakers with 30 ml of water and were exposed to increasing concentrations of piplartine or niclosamide until the EC_50_ was obtained. Four replicates per concentration were performed, totaling 20 neonates per condition. The negative control group consisted of 20 organisms exposed to cultivation water under the same experimental conditions used during the assays. After 48 h of exposure, the number of immobile organisms was observed and recorded. For the results of the experiment to be valid, up to 10% of the organisms in the negative control group were expected to be immobile. The Trimmed Spearman-Karber method [21] was used to calculate the median immobilization concentration (EC_50_), and the results were expressed in µg/ml.

#### Fish assays

Forty-eight hour static acute toxicity tests with *D. rerio* (zebrafish) were conducted according to a standard protocol [22]. The assays were performed in beakers containing 2,000 ml of synthetic soft water (pH 7.0–7.5, water hardness 40–48 mg/l CaCO_3_), maintained at 25±1°C with oxygenation under a 14 h light/10 h dark cycle. Ten fish were exposed to increasing dilutions of piplartine or niclosamide to determine the LC_50_ values. The mortality rate was recorded after 30 min, 24 h and 48 h, and the LC_50_ values were then calculated. Synthetic soft water containing 1% DMSO was used as a negative control.

#### Statistical analysis of the biological assays

The LC_90_ values were obtained by logistic regression adjustment using a log-dose transformation. The EC_50_ (immobilization) and LC_50_ (lethality) values and their 95% confidence limits were determined with the Trimmed Spearman-Karber method [21].

## Results

Extracts from the root, stem, leaf and fruit of *P. tuberculatum* had different molluscicidal activities, the root extracts was the most active, followed by fruit, stem and leaf extracts. Root extract was at least 15 times more active than extracts from other parts of *P. tuberculatum* (Table 1).

**Table 1:** Mortality of *B. glabrata* adults exposed to methanolic extracts of different organs of *Piper tuberculatum*.

*P. tuberculatum organ*	Concentration (µg/ml)								LC_50_ (µg/ml) [confidence interval]
	800	400	200	100	50	25	12.5	6.25	
Root	30	30	30	30	30	16	8	2	20.28 [16.65–24.69]
Stem	30	24	9	5	4	1	2	0	200.00 [161.37–247.87]
Leaf	30	21	4	3	1	3	2	2	310.27 [258.87–371.89]
Fruit	30	24	18	15	7	1	3	2	126.27 [97.08–164.25]

n = 30 adult snails.

Values were obtained at the end of the 7^th^ day of observation.

### PCA and PLS Analysis

A preliminary exploratory analysis was performed using PCA with 395 values of ion abundances from mass spectra data and 367 values of integrated areas from ^1^H NMR. The data from this analysis clustered into groups according to the organ of *P. tuberculatum* from which the extract was obtained. These data were then labeled according to their respective LC_50_ values. The scores plot generated from the ESIMS data (Figure S1) revealed a clear difference between roots and other organs (fruit, leaf and stem), which are grouped on the left side of the first principal component (PC1) and account for 81% of the total variance. The corresponding loadings plot shows that quasi-molecular ions with a *m/z* of 221, 222, 340 and 341 contribute significantly to this factor (Figure S1). These quasi-molecular ions correspond to the sodium adduct and ion fragments of piplartine (Figure S2). Additionally, the second principal component (PC2) explained 16% of the total variance and, together with the loadings plot, was used to assign quasi-molecular ions to pellitorine (*m/z* of 224 and 225), piperine (*m/z* of 286) and dihydropiperine (*m/z* of 288), which are characteristic components of the fruit. The amide dihydropiperlonguminine (*m/z* of 276) is responsible for the spatial separation between leaves and stems on the graph [10].

Scores and loadings plots from the PCA generated from integrated ^1^H NMR data (Figure S3) revealed that PC1 (which explained 89% of the total variance) discriminated between root extracts (the most active extract against *B. glabrata*; shown on the right side of the scores plot), fruit and stem extracts (grouped into the second quadrant, near the center) and leaf extracts (the least active, shown in the third quadrant). Importantly, the two first PCs explain nearly 100% of the total variance, and the separation of the roots from the other organ groups is largely due to methoxyl signals (δ 3.9) from piplartine (Figure S4). The fruits and stems were grouped according to the signals corresponding to the presence of pellitorine (δ 0.82–0.92 and δ 1.24–1.30).

**Figure 1:**
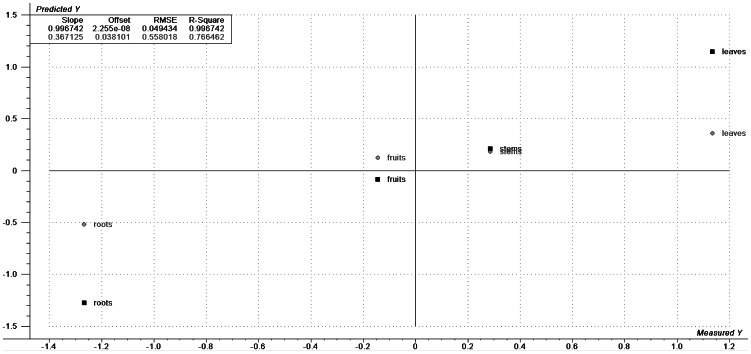
Calculated (squares) and predicted (circles) PLS data. These data were generated from ESIMS data, versus measured and autoscaled LC_50_ values of *P. tuberculatum* extracts for *B. glabrata*.

The PLS results from the ESIMS data generated significant coefficients of determination and values of internal prediction (0.98 and 0.83, respectively); however, only four samples were analyzed in this study. Figure 1 shows the experimental LC_50_ values and the predictions of the model. From the scores and correlation loadings plot in Figure S5, it was determined that quasi-molecular ions with *m/z* ratios of 221, 222, 340 and 341 (highly represented in root extracts) inversely correlate with their LC_50_ values, which indicate that these quasi-molecular ions are the major contributors to root extract activity.

**Figure 2:**
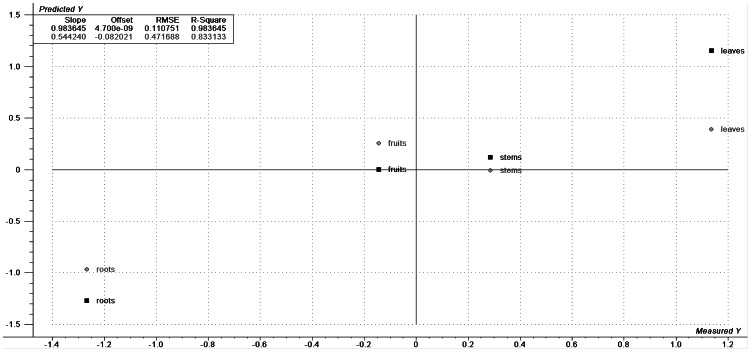
Calculated (squares) and predicted (circles) PLS data. These data were generated from ^1^H NMR data, versus measured and autoscaled LC_50_ values of *P. tuberculatum* extracts for *B. glabrata*.

PLS analysis of the ^1^H NMR data provides the coefficient of determination and values of internal prediction (0.98 and 0.83, respectively), which can be visualized on a plot of measured and predicted values of LC_50_ (Figure 2). This result, shown in the PLS analysis using ESIMS data, corroborates with the initial findings obtained by the PCA analysis. Using the scores plot and correlation loadings plot (Figure S6), was determined that the integrated regions with values of δ 0.86, 0.88, 0.9 and 3.89 have a negative correlation to their LC_50_ values; therefore, extracts with greater values of integrated regions (namely, root extracts) are more active.

### Isolation of Amides

Amides were isolated as crystals, and these amides were identified as piplartine, piperlonguminine, piperine and pellitorine by comparing their spectroscopic data with the literature [10].

### Molluscicidal and Ovicidal Activities

Piplartine, pellitorine, piperlonguminine and piperine were initially evaluated at 20 µg/ml against *B. glabrata* adults. While pellitorine, piperlonguminine and piperine were not active at this concentration (Table 2), piplartine caused 100% mortality following 24 h of treatment, and the same result was observed at 10, 9 and 8 µg/ml. Concentrations of 7, 6 and 5 µg/ml caused 86.6, 70.0 and 46.6% mortality, respectively, after 7 days. Thus, the LC_90_ and LC_50_ were 6.94 and 4.19 µg/ml, respectively (Table 3). The number of dead snails was not higher than 3.3% in the negative control group, which was included in the final statistical analysis.

**Table 2:** Mortality of *B. glabrata* adults and embryos exposed to amides (20 µg/ml).

Developmental Stage	Concentration(µg/ml)	Pellitorine		Piperine		Piperlonguminine		Piplartine	
		Dead (%)	n	Dead (%)	n	Dead (%)	n	Dead (%)	n
**Adult**	0^*^	1 (10)	10	0 (0)	10	0 (0)	10	0 (0)	10
	20	2 (20)	10	2 (20)	10	2 (20)	10	10 (100)	10
**Blastula**	0^*^	0 (0)	102	0 (0)	110	0 (0)	102	119 (100)	119
	20	0 (0)	131	0 (0)	111	0 (0)	114	122 (100)	122
**Gastrula**	0^*^	0 (0)	102	0 (0)	97	0 (0)	102	102 (100)	102
	20	0 (0)	91	0 (0)	90	0 (0)	125	134 (100)	134
**Trocophore**	0^*^	0 (0)	117	0 (0)	90	0 (0)	95	99 (100)	99
	20	0 (0)	122	0 (0)	129	0 (0)	147	103 (100)	103
**Veliger**	0^*^	0 (0)	109	0 (0)	103	0 (0)	100	119 (100)	119
	20	0 (0)	143	0 (0)	136	0 (0)	105	127 (100)	127

n = 10 snails for the adult stage; total embryo number for the other stages.

0^*^ = negative control (1% DMSO).

Values were obtained at the end of the 7^th^ day of observation.

**Table 3:** LC_50_ and LC_90_ for *B. glabrata* embryos and adults exposed to piplartine.

		Embryo stage				Adult
		Blastula	Gastrula	Trocophore	Veliger	
**piplartine**	**LC_50_ (µg/ml)**	0.64 [0.60–0.68]	1.75 [1.67–1.84]	2.83 [2.77–2.89]	3.73 [3.66–3.79]	4.19 [4.01–4.37]
	**LC_90_ (µg/ml)**	0.99 [0.93–1.06]	2.50 [2.40–2.63]	3.33 [3.26–3.43]	4.35 [4.26–4.49]	6.94 [6.83–7.05]
**niclosamide**	**LC_50_ (µg/ml)**	nc	nc	nc	nc	0.05 [0.04–0.06]
	**LC_90_ (µg/ml)**	nc	nc	nc	nc	0.09 [0.08–0.09]

[ ] 95% confidence interval.

nc – not calculated.

**Figure 3:**
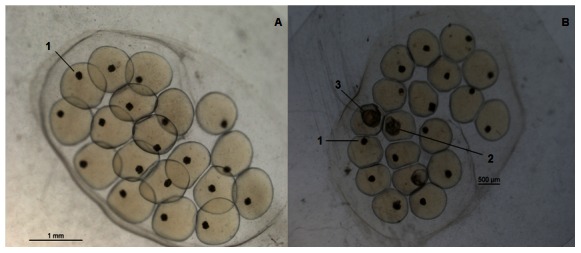
*B. glabrata* embryos exposed to piplartine at the blastula stage. **A**) Immediately following exposure to 1.2 µg/ml piplartine, **B**) during the 7 day observation period after exposure to 1.0 µg/ml piplartine (1- dead, 2 - normal, 3 – malformed).

Piplartine, pellitorine, piperlonguminine and piperine were evaluated at a concentration of 20 µg/ml against the blastula, gastrula, trocophore and veliger stages. Piplartine was the only amide that caused 100% mortality to embryos at all stages. Thus, piplartine was evaluated in a dose-response assay; interestingly, sensitivity was inversely correlated with the developmental stage: the LC_100_ was 1.2 ppm for the blastula stage (Figure 3), 2.2 µg/ml for the gastrula, 3.6 µg/ml for the trocophore and 5.0 µg/ml for the veliger stage. The mortality rate did not exceed 2% in the embryonic negative control group for any stage.

### Toxicity of Piplartine

**Figure 4:**
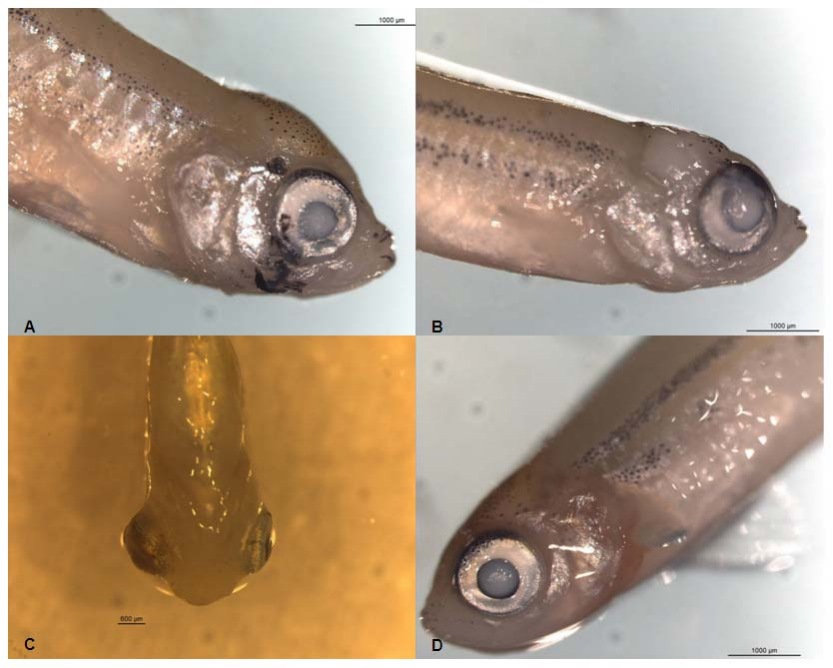
Morphological changes in *D. rerio* during the 48 hours of exposure to piplartine. **A**) Leakage of the ocular pigment caused by 1.8 ppm piplartine, **B**) tissue alterations on the head and mouth caused by 1.6 µg/ml piplartine, **C**) exophthalmia and hemorrhage caused by 1.4 µg/ml piplartine and D) control group.

Given the effectiveness of piplartine as a molluscicide and ovicide, the acute toxicity of the compound to *D. similis* and *D. rerio* was investigated (Table 4). Piplartine was nearly five times more toxic to *D. rerio* than to *D. similis*. Lethality and immobilization were the endpoints applied to estimate LC_50_ to *D. rerio* and *D. similis*, respectively. General abnormalities were also recorded during the *D. rerio* experiments, such as erratic swimming, extended abdomen, body hemorrhaging, red pigmented spots, exophthalmia and abnormal head shape (Figure 4). These effects were transient and only occurred during the 48 h exposure period.

**Table 4:** Toxicity of piplartine and niclosamide to the freshwater microcrustacean *D. similis* and the fish *D. rerio*.

Species	Endpoint	Concentration (µg/ml)		Toxicity classification	
		piplartine	niclosamide	piplartine	niclosamide
*Daphnia similis*	Immobilization				
	24 h EC_50_	7.84 [7.46–8.15]	0.28 [0.23–0.30]		
	48 h EC_50_	7.32 [6.93–7.69]	0.25 [0.19–0.27]	Cat. 2	Cat. 1
*Danio rerio*	Lethality				
	24 h LC_50_	2.0 [1.87–2.13]	0.14 [0.11–0.17]		
	48 h LC_50_	1.69 [1.61–1.77]	0.12 [0.10–0.19]	Cat. 2	Cat. 1

Data are presented as EC_50_ or LC_50_ (µg/ml) with the respective 95% confidence limits in brackets.

Toxicity classification: Cat. 1 - acute toxicity ≤1.00 µg/ml; Cat. 2 - acute toxicity >1.00 but ≤10.0 µg/ml; Cat. 3 - acute toxicity >10.0 but <100 µg/ml [28].

## Discussion

Former studies indicated the root extract of *P. tuberculatum* as most potent among the extract from different parts of this plant. The multivariated analysis using NMR and MS data indicated the influence of the compounds present in the extracts on the molluscicidal activity.

The abundance of quasi-molecular ions with *m/z* ratios of 340 and 221 (ESIMS data) and a δ 3.89 signal (^1^H NMR spectra) corresponding to the amide piplartine, noted for its molluscicidal activity. Indeed, piplartine has a wide range of biological activities, including cytotoxicity against cultured tumor cells and antiproliferative, anti-platelet aggregation, antifungal, insecticidal, trypanocidal, leishmanicidal and schistosomicidal properties [14], [16], [23]. Piplartine exhibited molluscicidal and ovicidal activities at a concentration lower than the concentration recommended by the WHO for a molluscicide candidate (activity at less than 20 ppm). The amide was approximately seven times more toxic to embryos than to adult snails (LC_90_ of 0.99 µg/ml and 6.94 µg/ml, respectively); additionally, embryos at the blastula stage were the most sensitive to piplartine, followed by the gastrula, trocophore and veliger stages. Embryos in the early stages of development are mitotically very active and are expected to exhibit a higher sensitivity to chemical compounds [12], [24], [25]. In addition, embryos exposed to concentrations below the LC_100_ had malformations, particularly when exposed to the compound in the initial stages of development, namely, the blastula and gastrula stages. The death of the embryos is likely related to the induction of embryonic malformations because embryos with such malformations generally show delayed embryonic development and die during the spawning stage [26], [27] (Figure 3B).

Despite its potential molluscicidal activity, piplartine is classified as a category 2 toxin to *D. similis* and *D. rerio* according to the Global Harmonization System [28] and a category 3 toxin (LD_50_ 32.3±3.4 µg/ml) to *Artemia salina* [29]; the compound is, however, substantially less toxic than niclosamide (category 1) to *D. similis* and *D. rerio* organisms.

These results implicate piplartine as a potential natural molluscicide that acts by interfering with the life cycle of the parasitic trematode *S. mansoni* by eradicating the parasite's intermediate host. Piplartine not only efficiently kills adults of *B. glabrata* at low concentrations (LC_50_ 4.19 µg/ml) but also leads to the lethality of the embryos inside the eggs, minimizing recolonization of the environment by the mollusks.

## Supporting Information

Figure S1 **Scores and loadings plots from PCA generated using ESIMS data of** ***P. tuberculatum*** **extracts.** Samples, which represent extracts, are labeled according to their respective activities: more activity – dot, intermediate activity – triangle and less activity – square.
(TIF)

Figure S2 **HRESI mass spectrum of piplartine with [M+Na]^+^ = 340.1128 and its fragmentation.**
(TIF)

Figure S3 **Scores and loadings plots from PCA, generated using ^1^H NMR data, on** ***P. tuberculatum*** **extracts.** Samples, which represent the various extracts, are labeled according to their respective activities: more activity – dot, intermediate activity – triangle and less activity – square.
(TIF)

Figure S4 **NMR spectrum of piplartine (200 MHz, Bruker).**
(TIF)

Figure S5 **Scores and weights plot generated by PLS analysis.** These data were obtained using autoscaled LC_50_ values for *B. glabrata* and spectra signals from integrated regions using ^1^H NMR on *P. tuberculatum* extracts.
(TIF)

Figure S6 **Scores and weights plot generated by PLS analysis.** These data were obtained using autoscaled LC_50_ values for *B. glabrata* and ion abundances from *P. tuberculatum* extracts.
(TIF)
