# Supplementary figures and images for: Schistosomiasis Control Using Piplartine against Biomphalaria glabrata at Different Developmental Stages
Source: PLoS Negl Trop Dis. 2013 Jun 6;7(6):e2251. doi: 10.1371/journal.pntd.0002251 (PMC3675008; doi:10.1371/journal.pntd.0002251)

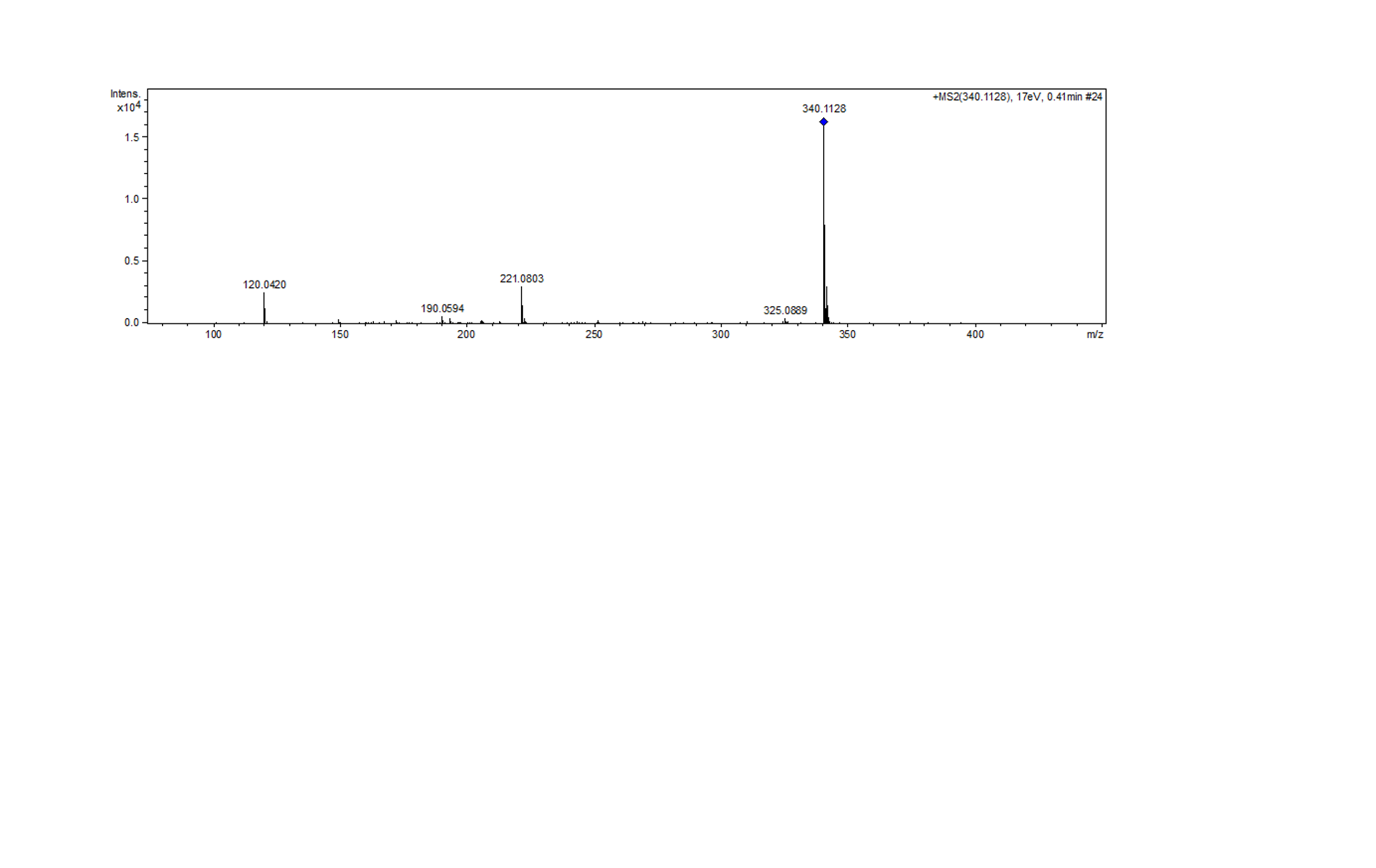

Supplement: Figure S2 — HRESI mass spectrum of piplartine with [M+Na]+ = 340.1128 and its fragmentation. (TIF) [file pntd.0002251.s002.tif]

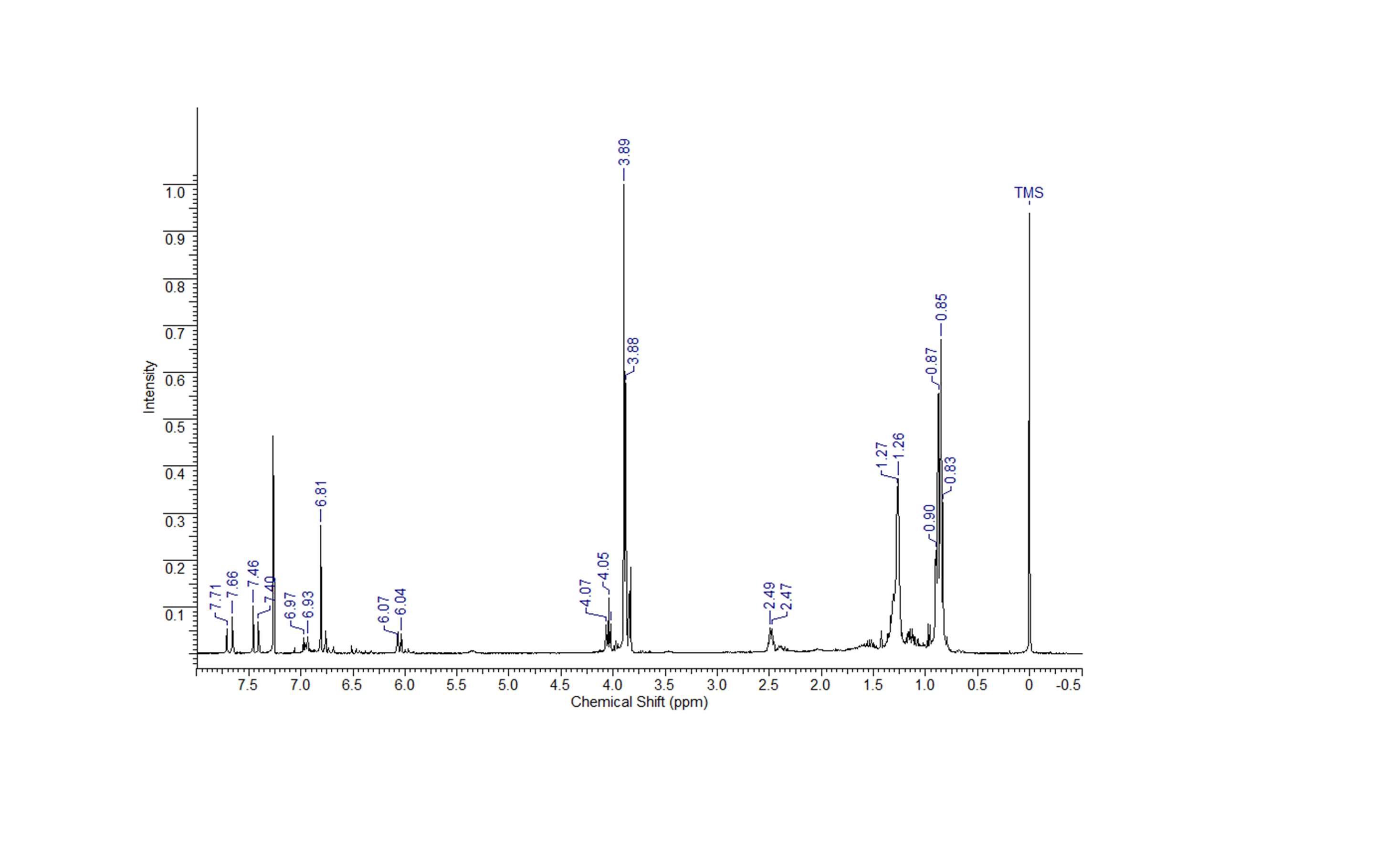

Supplement: Figure S4 — NMR spectrum of piplartine (200 MHz, Bruker). (TIF) [file pntd.0002251.s004.tif]
